# Supplementary material for: MHC class I and MHC class II reporter mice enable analysis of immune oligodendroglia in mouse models of multiple sclerosis
Source: eLife. 2023 Apr 14;12:e82938. doi: 10.7554/eLife.82938 (PMC10181822; doi:10.7554/eLife.82938)
Supplement: Figure 1—source data 3. [file elife-82938-fig1-data3.pdf]

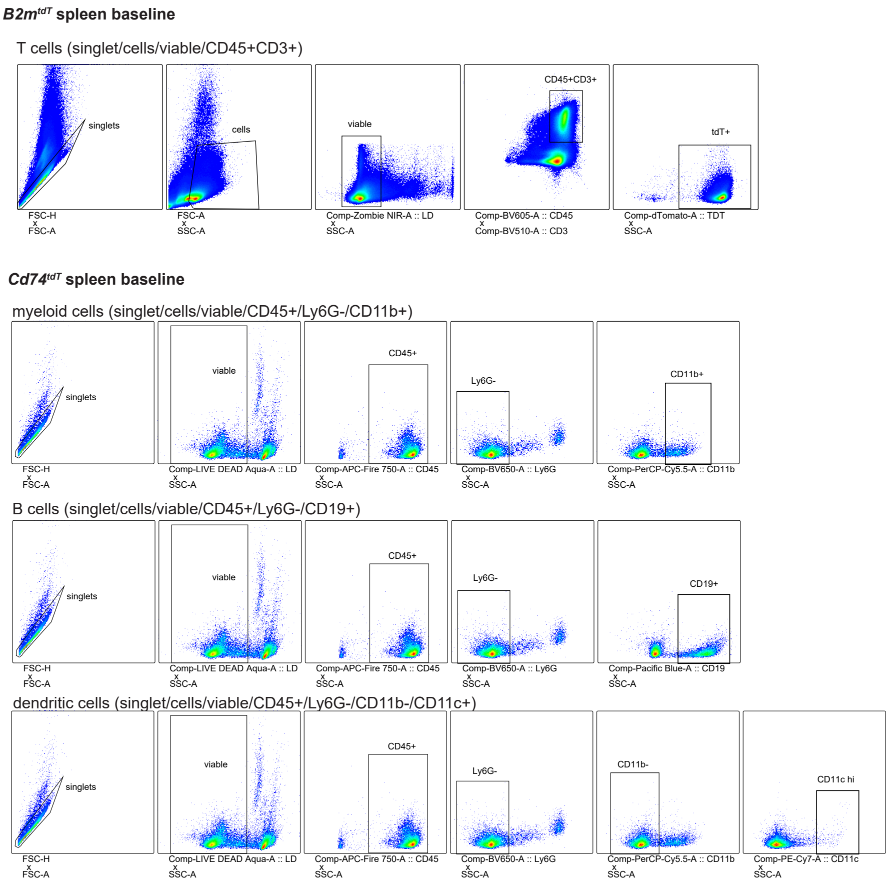

B2m^tdT spleen baseline
T cells (singlet/cells/viable/CD45+CD3+)
singlets
FSC-H x FSC-A
cells
FSC-A x SSC-A
viable
Comp-Zombie NIR-A :: LD x SSC-A
CD45+CD3+
Comp-BV605-A :: CD45 x Comp-BV510-A :: CD3
tdT+
Comp-dTomato-A :: TDT x SSC-A
Cd74^tdT spleen baseline
myeloid cells (singlet/cells/viable/CD45+/Ly6G-/CD11b+)
singlets
FSC-H x FSC-A
viable
Comp-LIVE DEAD Aqua-A :: LD x SSC-A
CD45+
Comp-APC-Fire 750-A :: CD45 x SSC-A
Ly6G-
Comp-BV650-A :: Ly6G x SSC-A
CD11b+
Comp-PerCP-Cy5.5-A :: CD11b x SSC-A
B cells (singlet/cells/viable/CD45+/Ly6G-/CD19+)
singlets
FSC-H x FSC-A
viable
Comp-LIVE DEAD Aqua-A :: LD x SSC-A
CD45+
Comp-APC-Fire 750-A :: CD45 x SSC-A
Ly6G-
Comp-BV650-A :: Ly6G x SSC-A
CD19+
Comp-Pacific Blue-A :: CD19 x SSC-A
dendritic cells (singlet/cells/viable/CD45+/Ly6G-/CD11b-/CD11c+)
singlets
FSC-H x FSC-A
viable
Comp-LIVE DEAD Aqua-A :: LD x SSC-A
CD45+
Comp-APC-Fire 750-A :: CD45 x SSC-A
Ly6G-
Comp-BV650-A :: Ly6G x SSC-A
CD11b-
Comp-PerCP-Cy5.5-A :: CD11b x SSC-A
CD11c hi
Comp-PE-Cy7-A :: CD11c x SSC-A
