# Supplementary figures and images for: MHC class I and MHC class II reporter mice enable analysis of immune oligodendroglia in mouse models of multiple sclerosis
Source: eLife. 2023 Apr 14;12:e82938. doi: 10.7554/eLife.82938 (PMC10181822; doi:10.7554/eLife.82938)

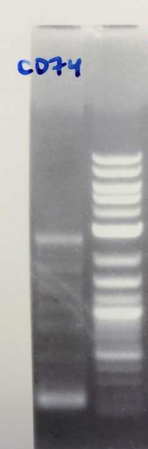

Supplement: Figure 1—source data 4. [file elife-82938-fig1-data4.zip › Cd74.png]

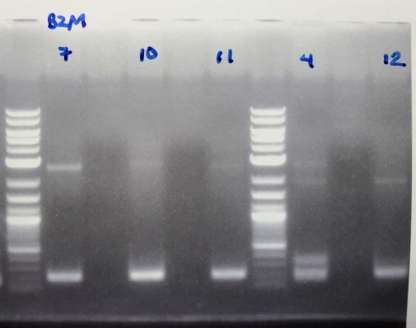

Supplement: Figure 1—source data 4. [file elife-82938-fig1-data4.zip › B2m.png]

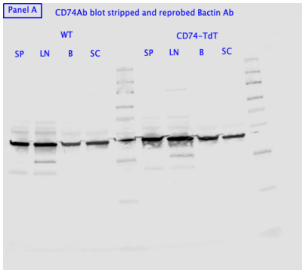

0004348\_02

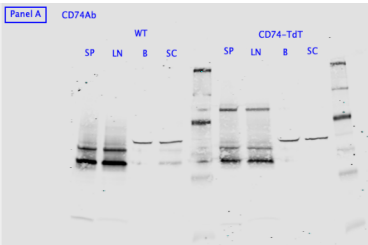

0004316\_02

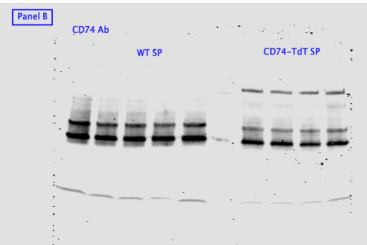

0004306\_02

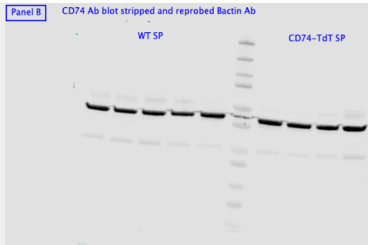

0004311\_02

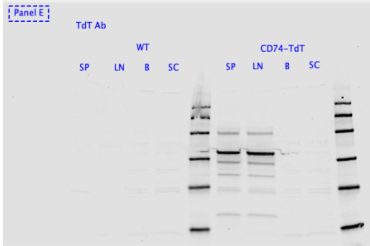

0004317\_03

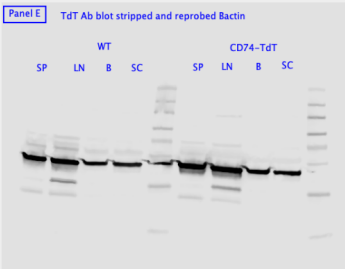

0004347\_05

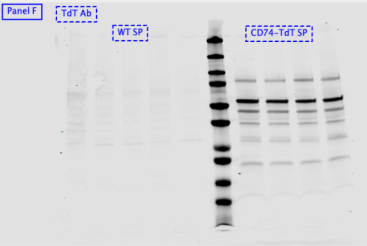

0004307\_02

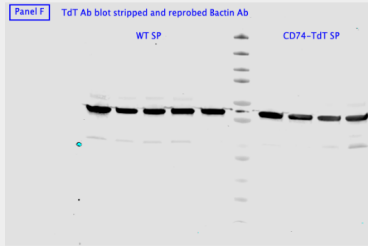

0004313\_02

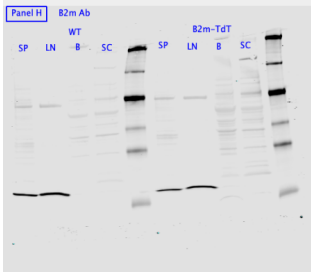

0004323\_02

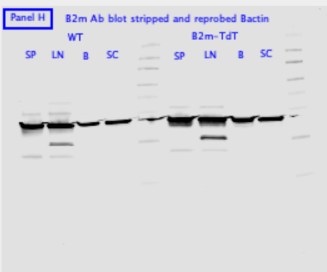

0004335\_02

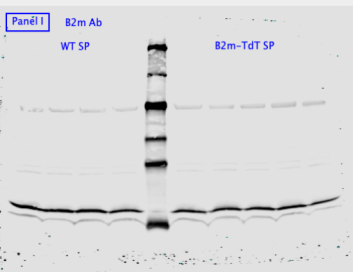

0004288\_03

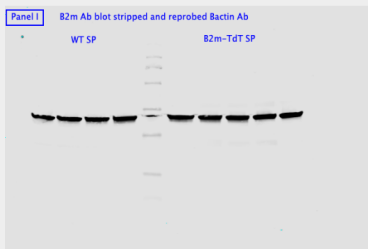

0004300\_03

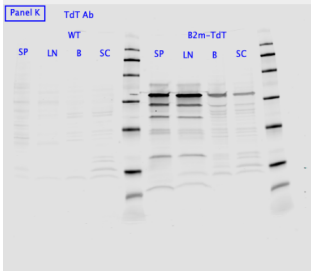

0004324\_02

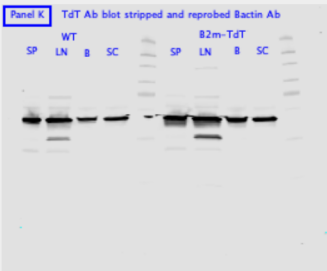

0004336\_02

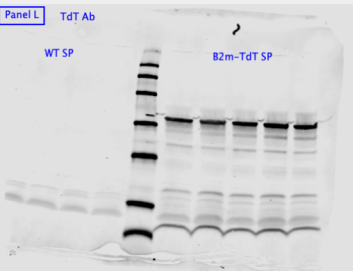

0004290\_03

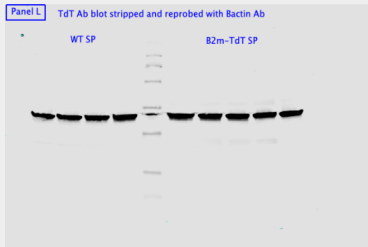

0004300\_04

Supplement: Figure 1—figure supplement 1—source data 1. — Blots are labeled with antibody probe, samples and upper left letter corresponds to panel depicted in Figure 1—figure supplement 1. [file elife-82938-fig1-figsupp1-data1.zip › Figure 1 figure supplement 1 source data 1.pdf]

# Spinal cord EAE

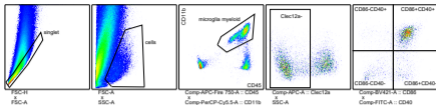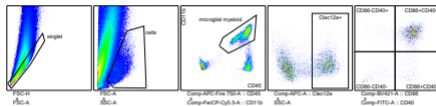

# Brain EAE

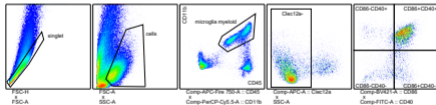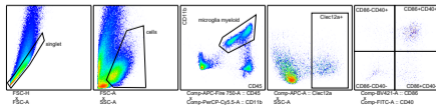

Supplement: Figure 6—source data 2. [file elife-82938-fig6-data2.pdf]

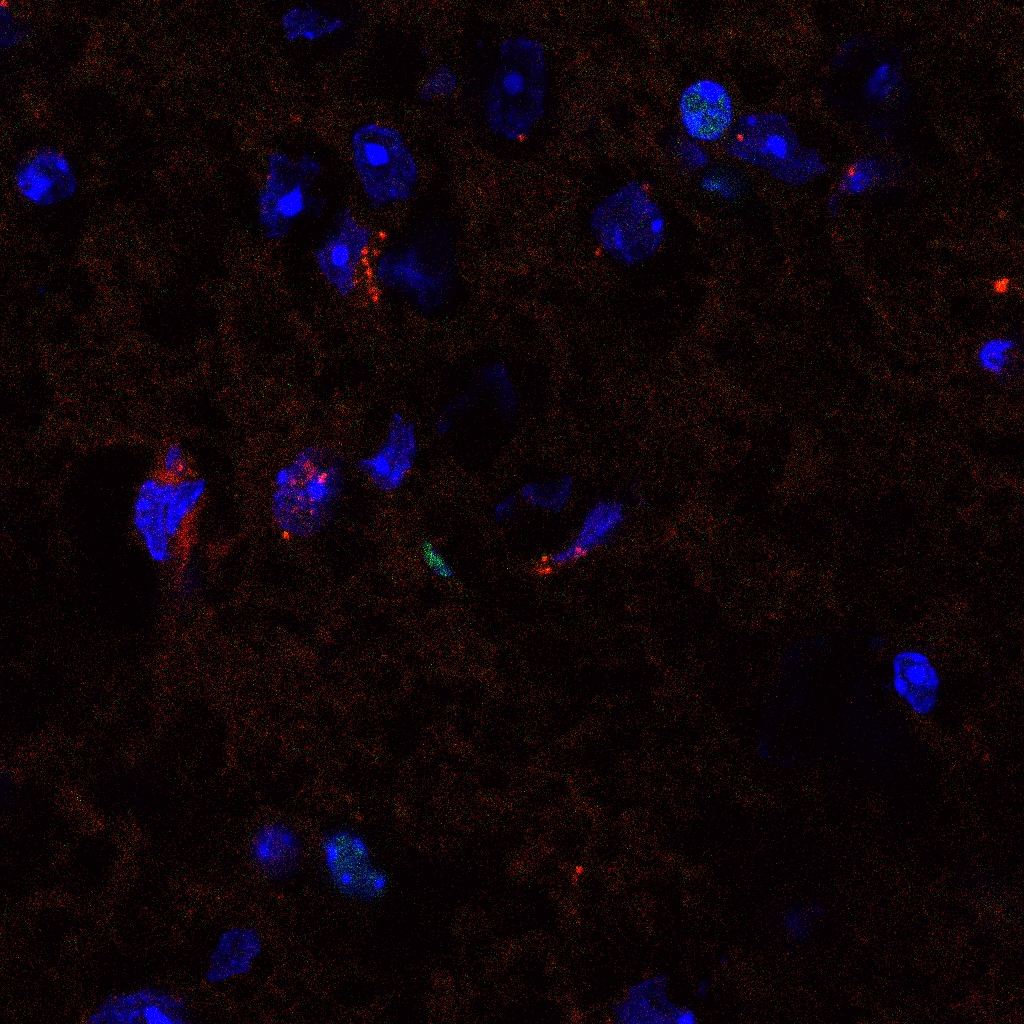

Supplement: Figure 8—source data 1. [file elife-82938-fig8-data1.zip › figure 8- source data 1/figure 8- source data 1_z17c1-3.jpg]

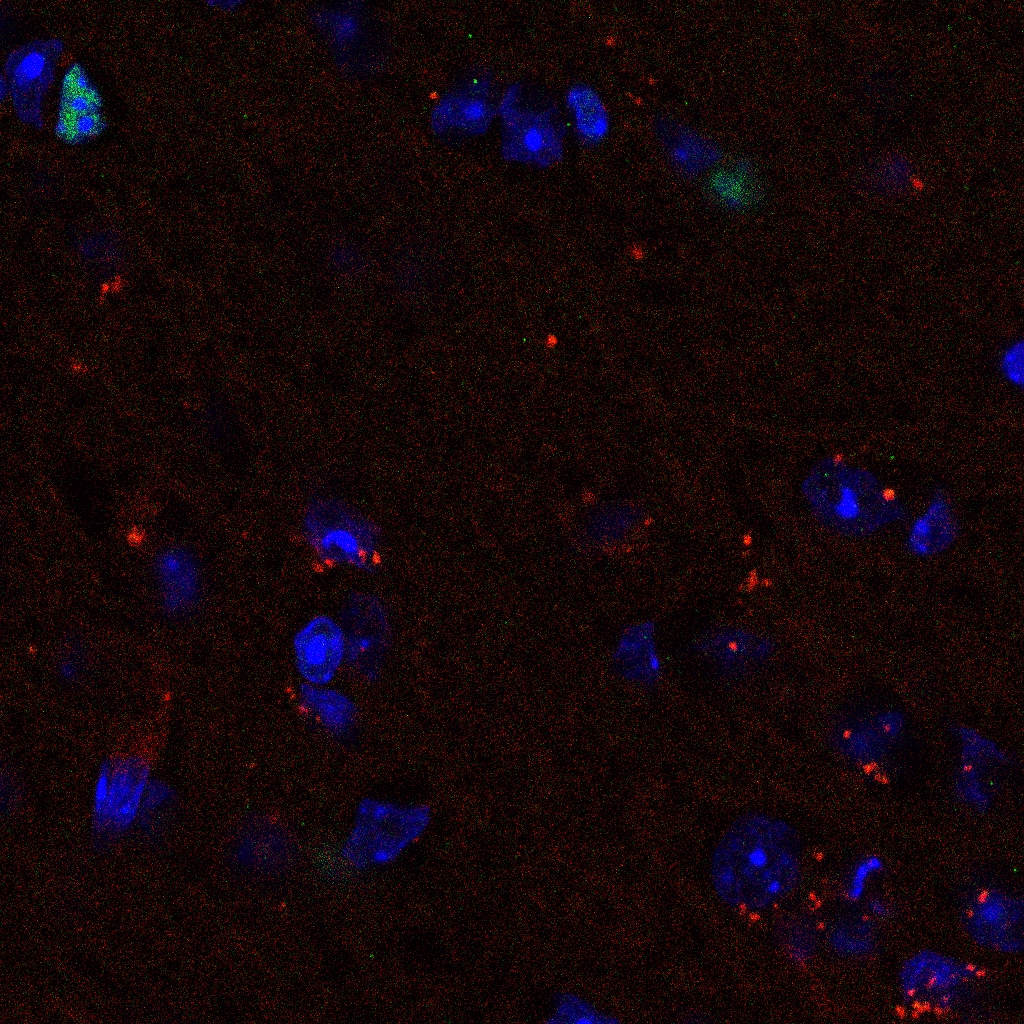

Supplement: Figure 8—source data 1. [file elife-82938-fig8-data1.zip › figure 8- source data 1/figure 8- source data 1_z05c1-3.jpg]

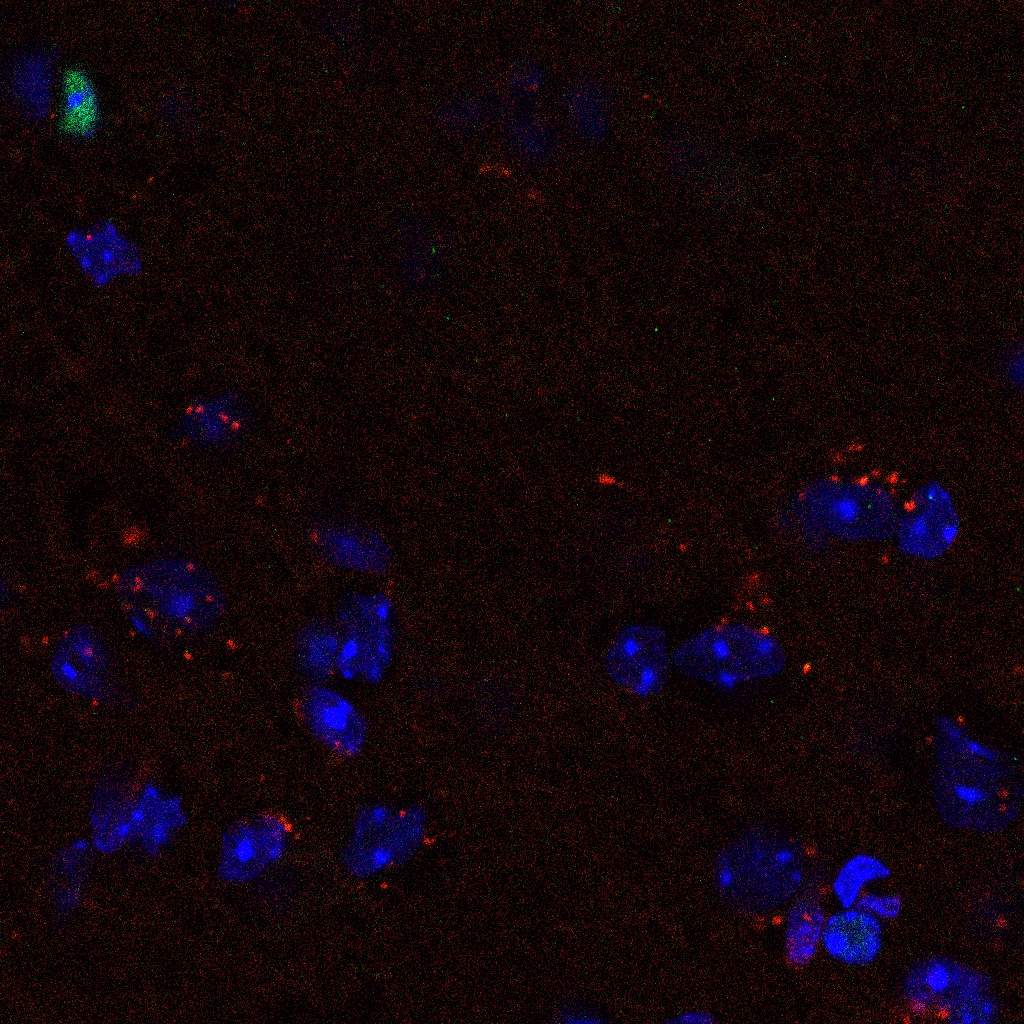

Supplement: Figure 8—source data 1. [file elife-82938-fig8-data1.zip › figure 8- source data 1/figure 8- source data 1_z01c1-3.jpg]

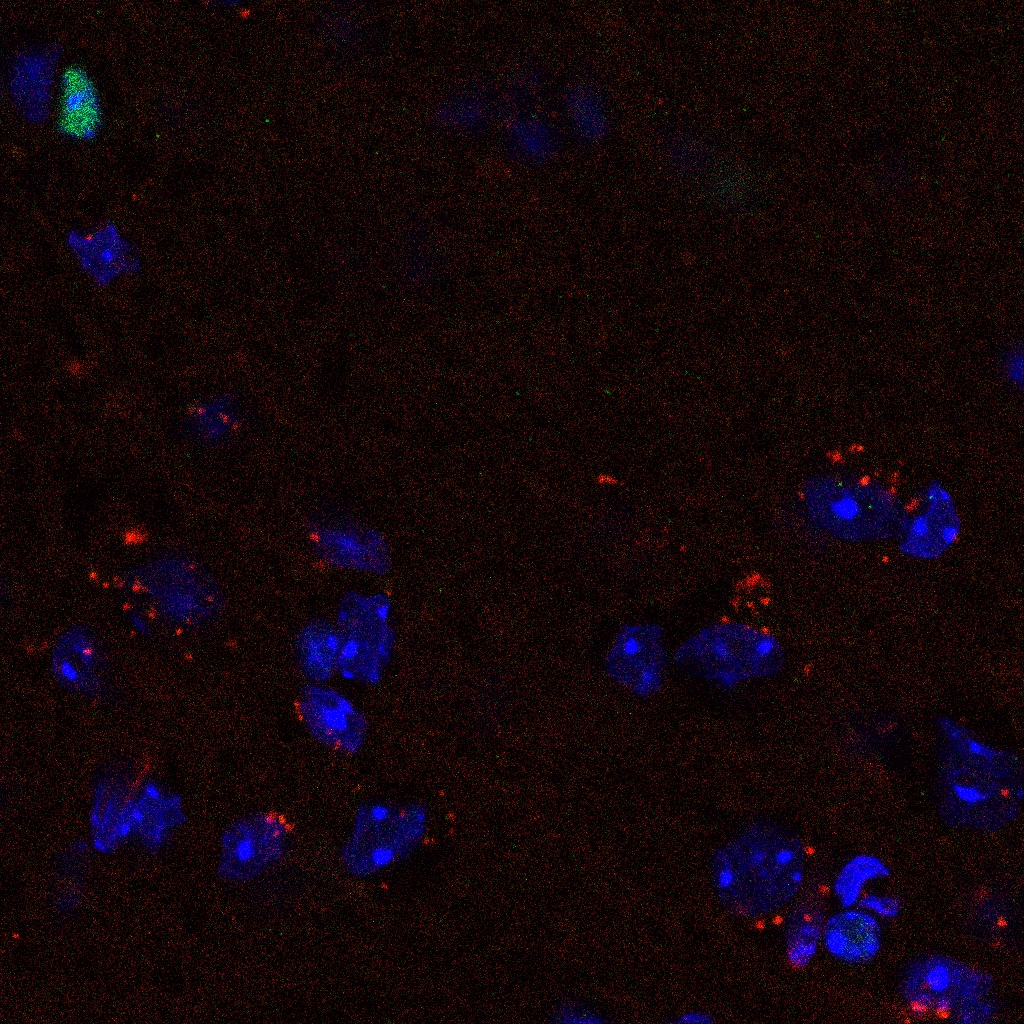

Supplement: Figure 8—source data 1. [file elife-82938-fig8-data1.zip › figure 8- source data 1/figure 8- source data 1_z02c1-3.jpg]

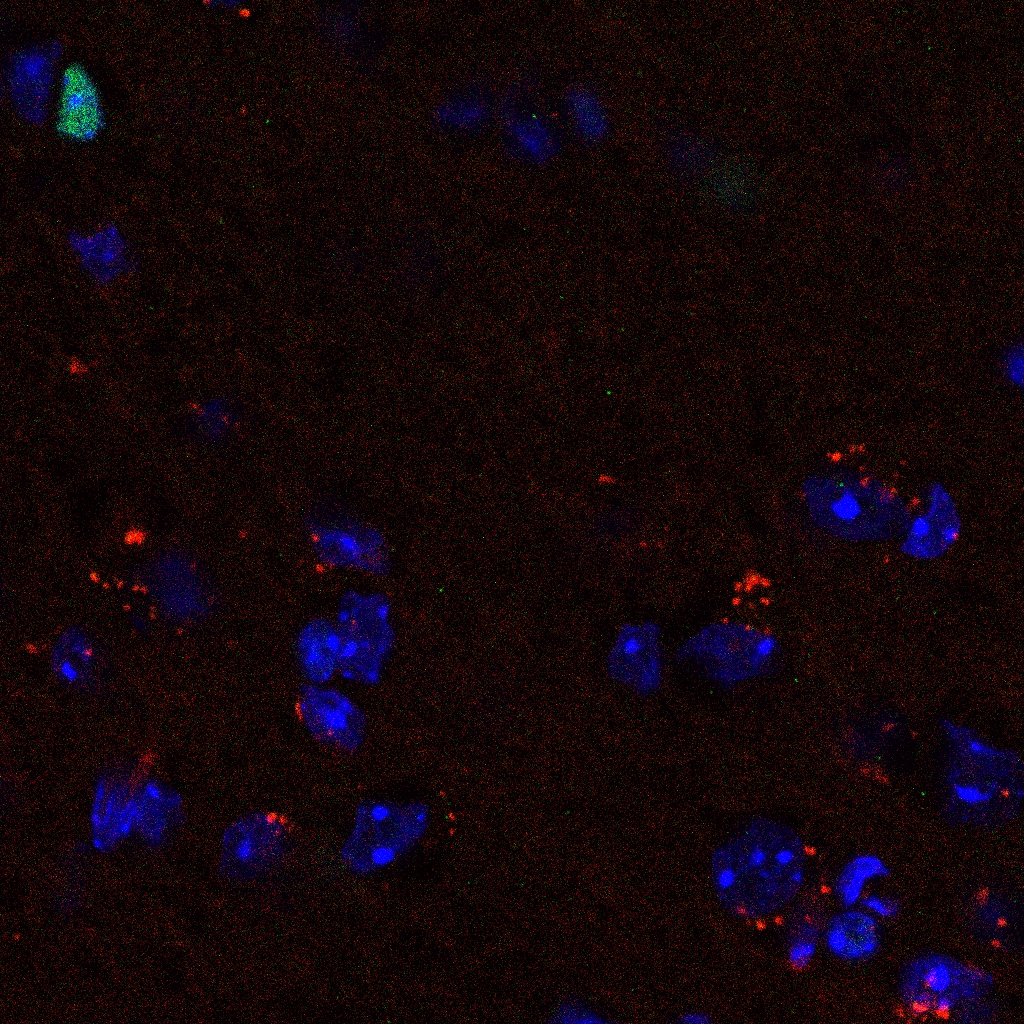

Supplement: Figure 8—source data 1. [file elife-82938-fig8-data1.zip › figure 8- source data 1/figure 8- source data 1_z03c1-3.jpg]

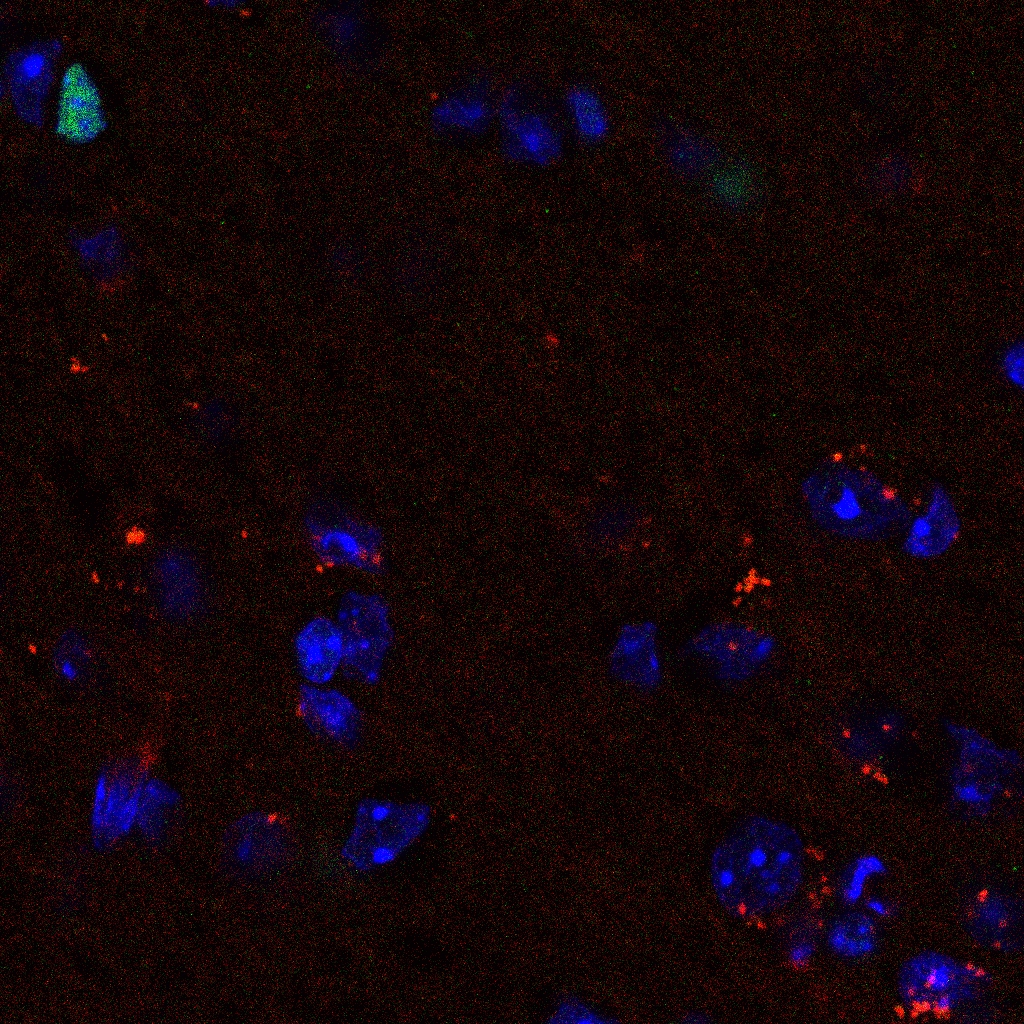

Supplement: Figure 8—source data 1. [file elife-82938-fig8-data1.zip › figure 8- source data 1/figure 8- source data 1_z04c1-3.jpg]

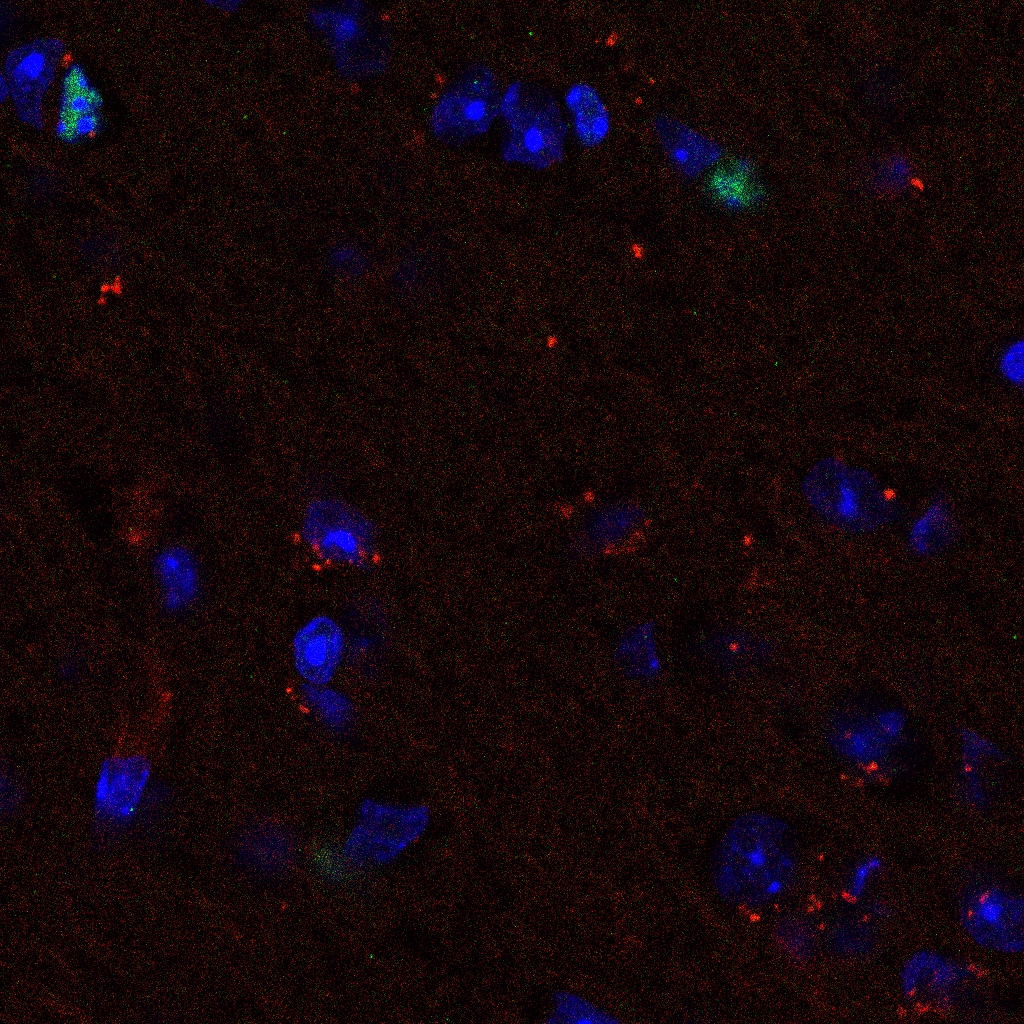

Supplement: Figure 8—source data 1. [file elife-82938-fig8-data1.zip › figure 8- source data 1/figure 8- source data 1_z06c1-3.jpg]

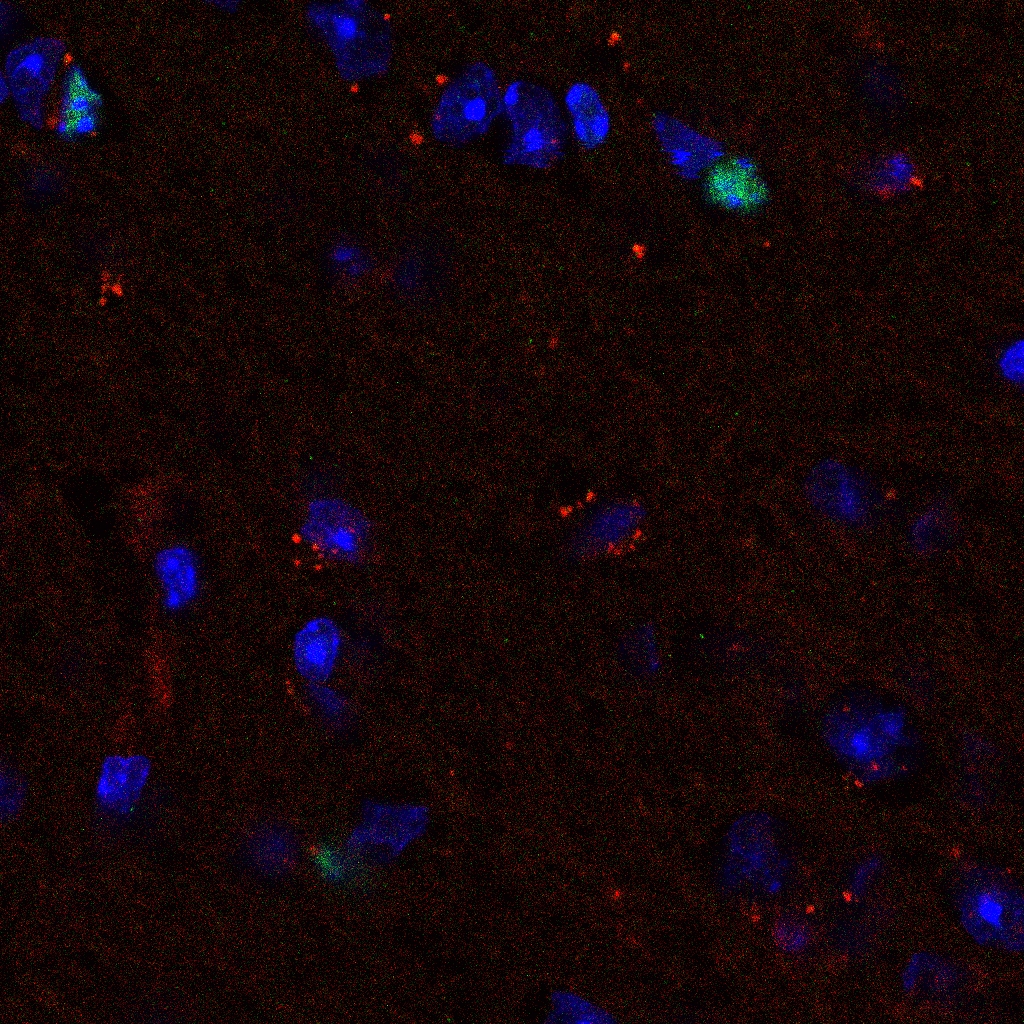

Supplement: Figure 8—source data 1. [file elife-82938-fig8-data1.zip › figure 8- source data 1/figure 8- source data 1_z07c1-3.jpg]

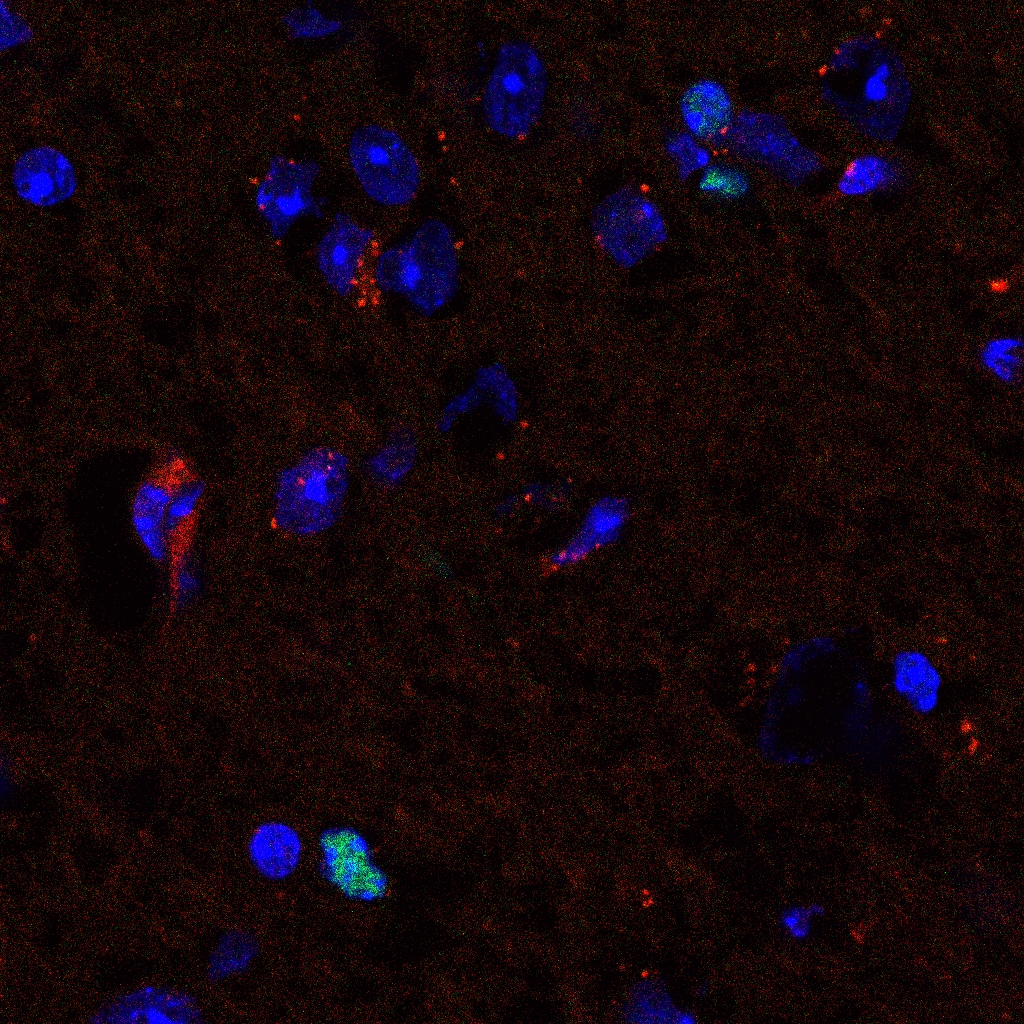

Supplement: Figure 8—source data 1. [file elife-82938-fig8-data1.zip › figure 8- source data 1/figure 8- source data 1_z15c1-3.jpg]

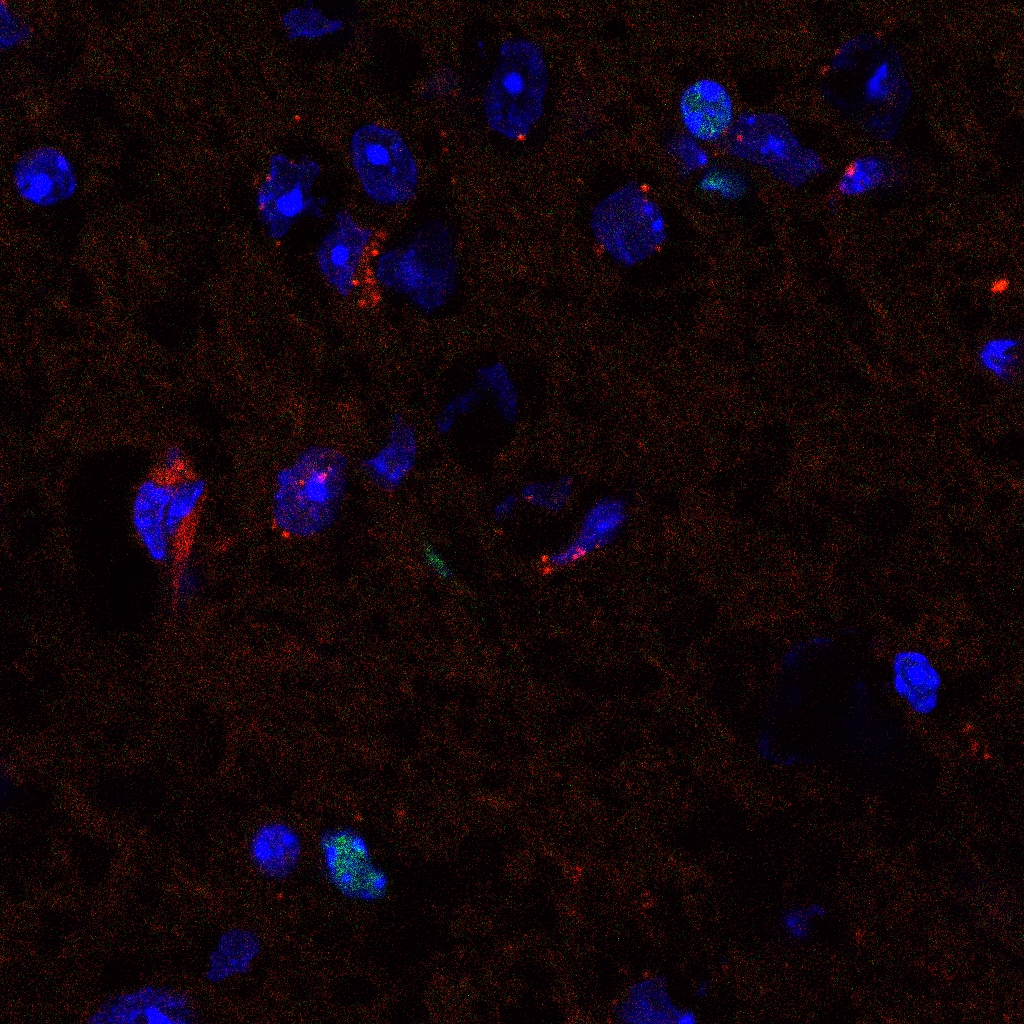

Supplement: Figure 8—source data 1. [file elife-82938-fig8-data1.zip › figure 8- source data 1/figure 8- source data 1_z16c1-3.jpg]

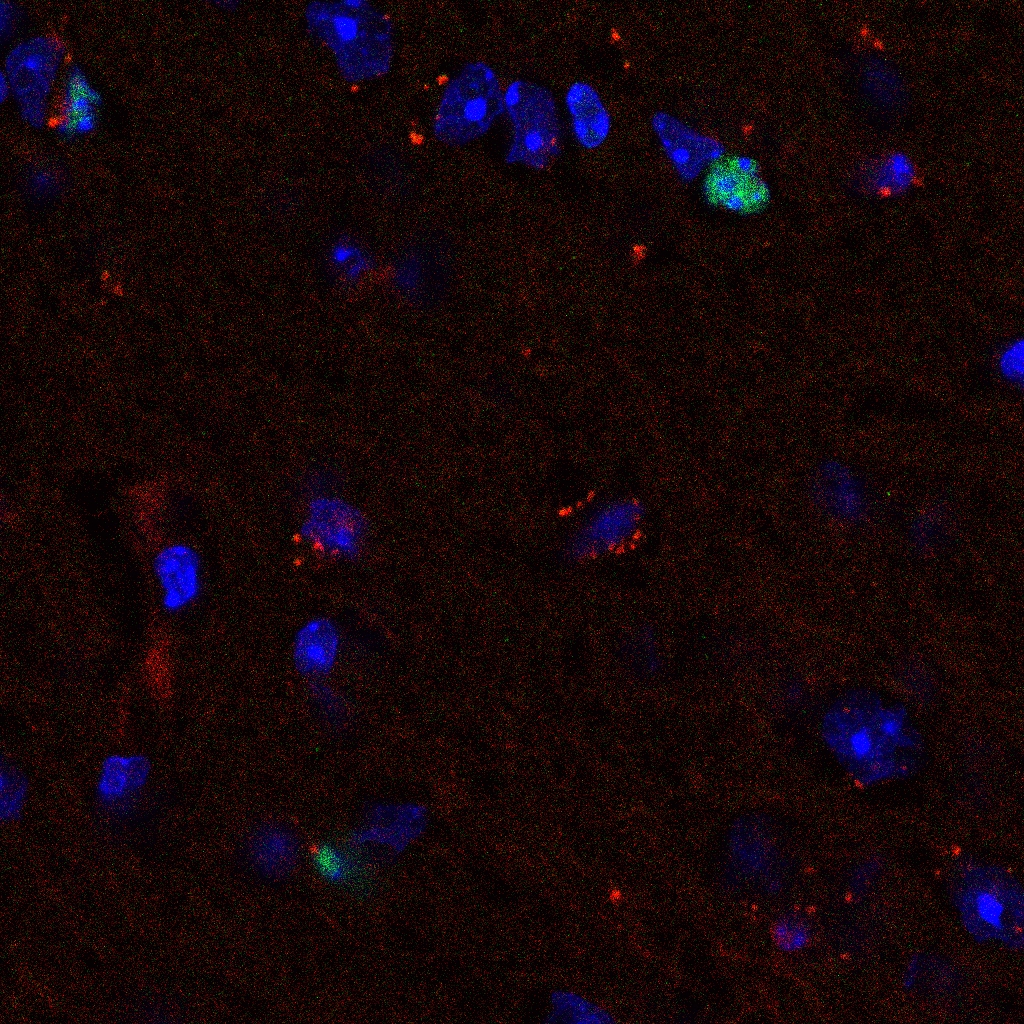

Supplement: Figure 8—source data 1. [file elife-82938-fig8-data1.zip › figure 8- source data 1/figure 8- source data 1_z08c1-3.jpg]

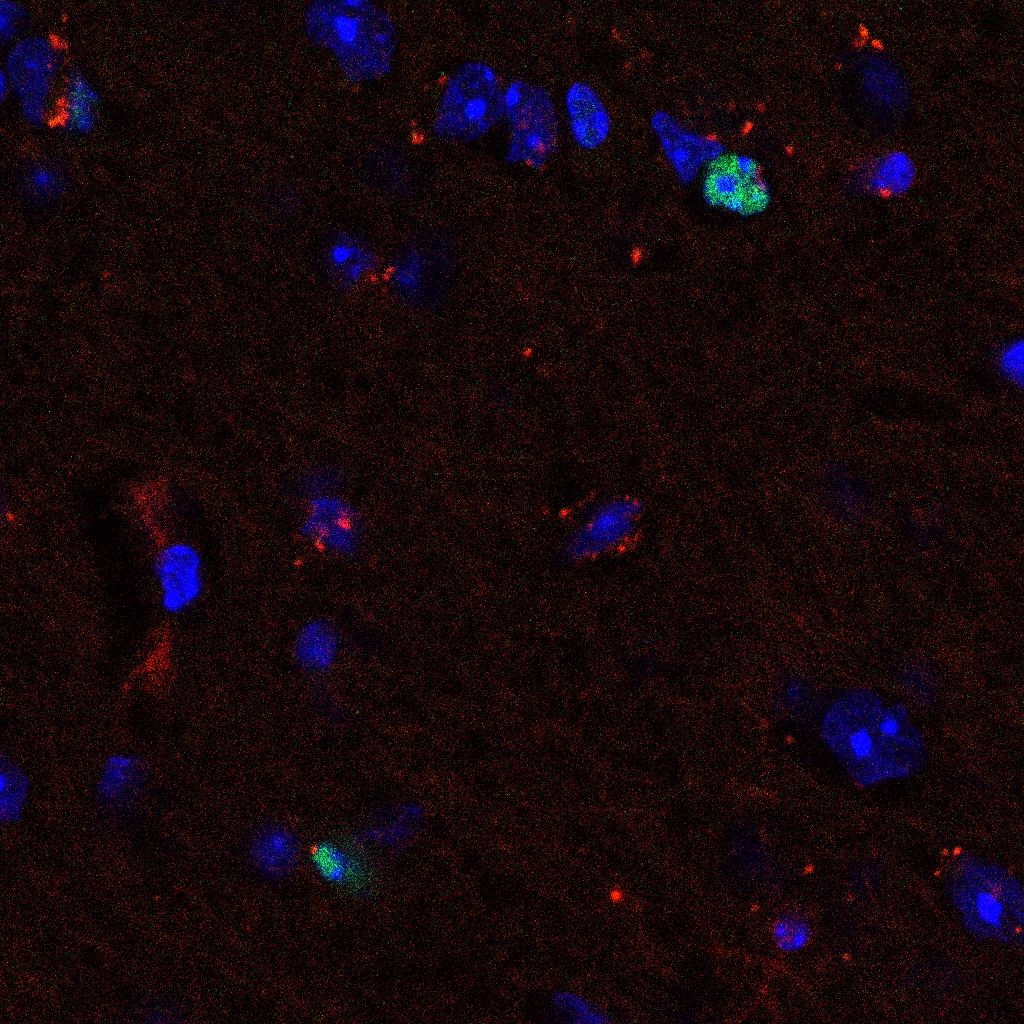

Supplement: Figure 8—source data 1. [file elife-82938-fig8-data1.zip › figure 8- source data 1/figure 8- source data 1_z09c1-3.jpg]

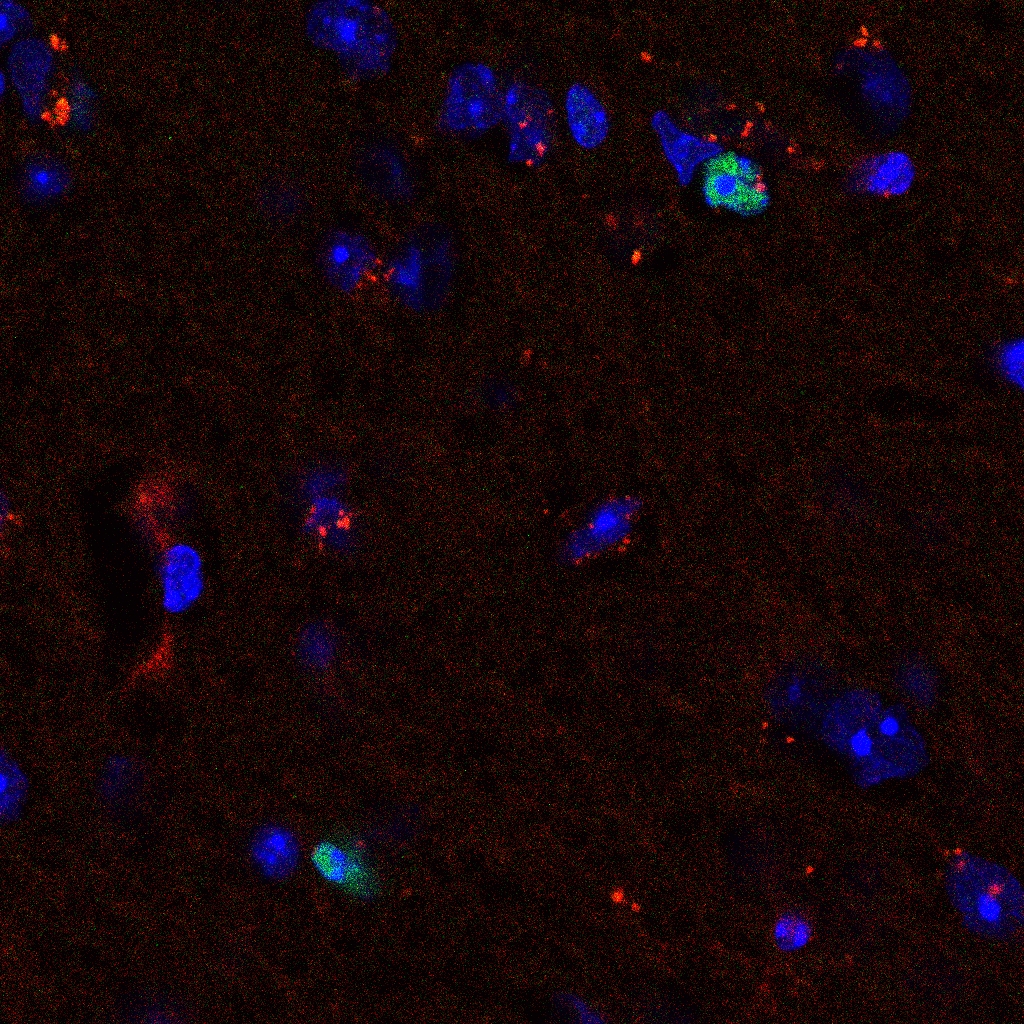

Supplement: Figure 8—source data 1. [file elife-82938-fig8-data1.zip › figure 8- source data 1/figure 8- source data 1_z10c1-3.jpg]

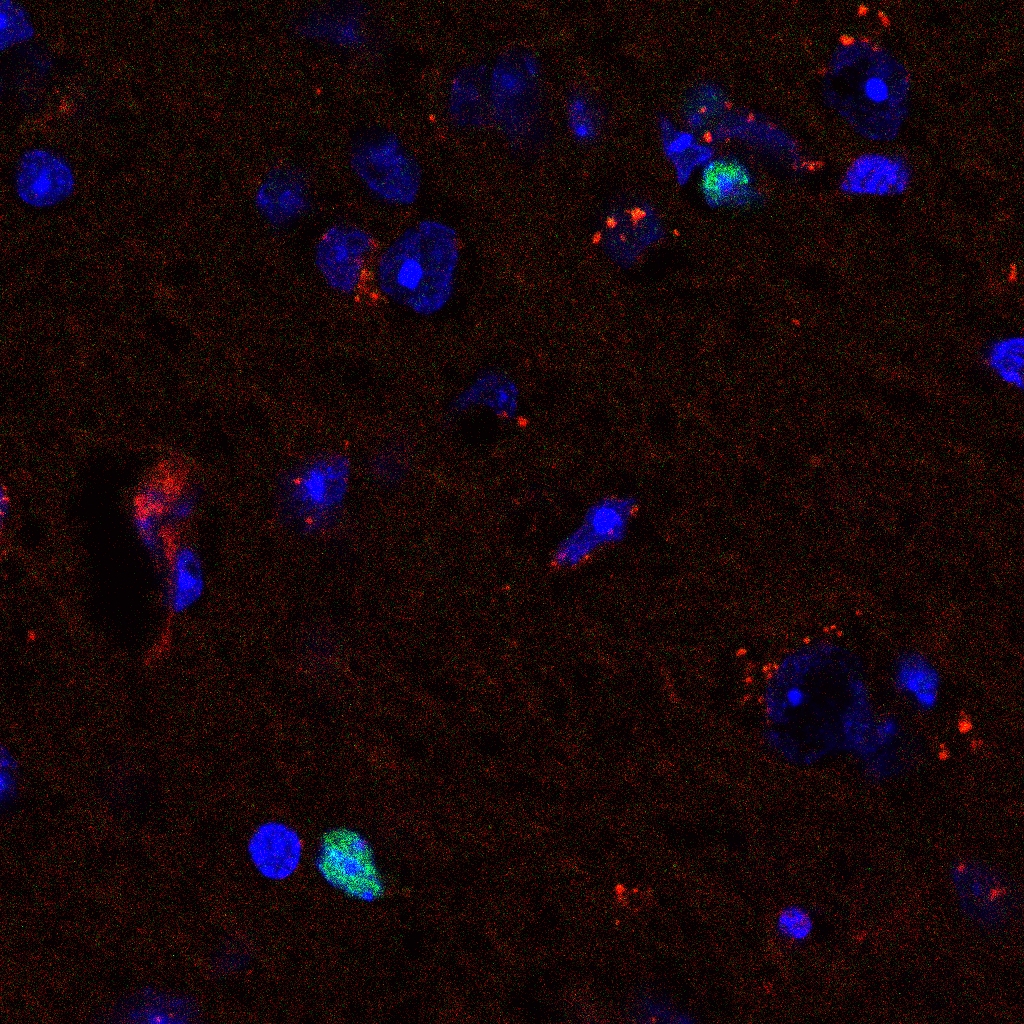

Supplement: Figure 8—source data 1. [file elife-82938-fig8-data1.zip › figure 8- source data 1/figure 8- source data 1_z13c1-3.jpg]

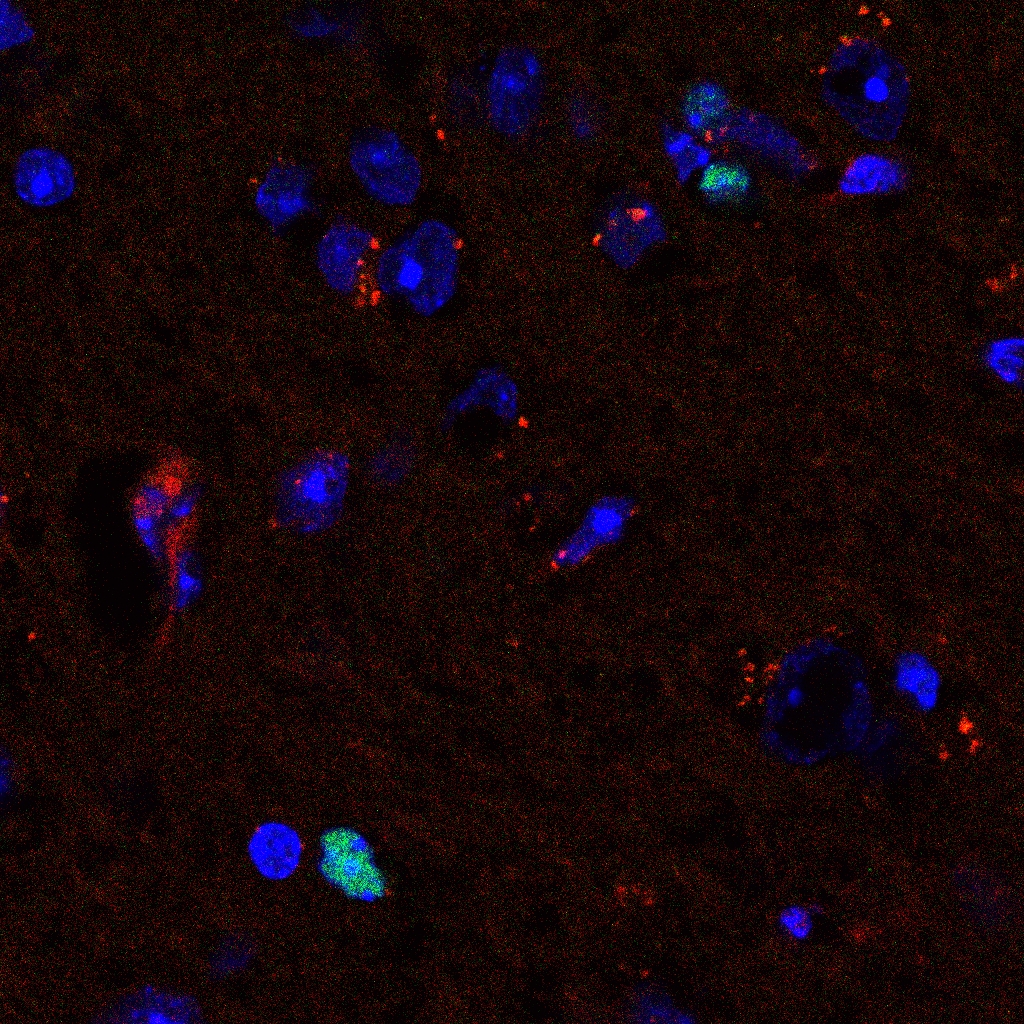

Supplement: Figure 8—source data 1. [file elife-82938-fig8-data1.zip › figure 8- source data 1/figure 8- source data 1_z14c1-3.jpg]

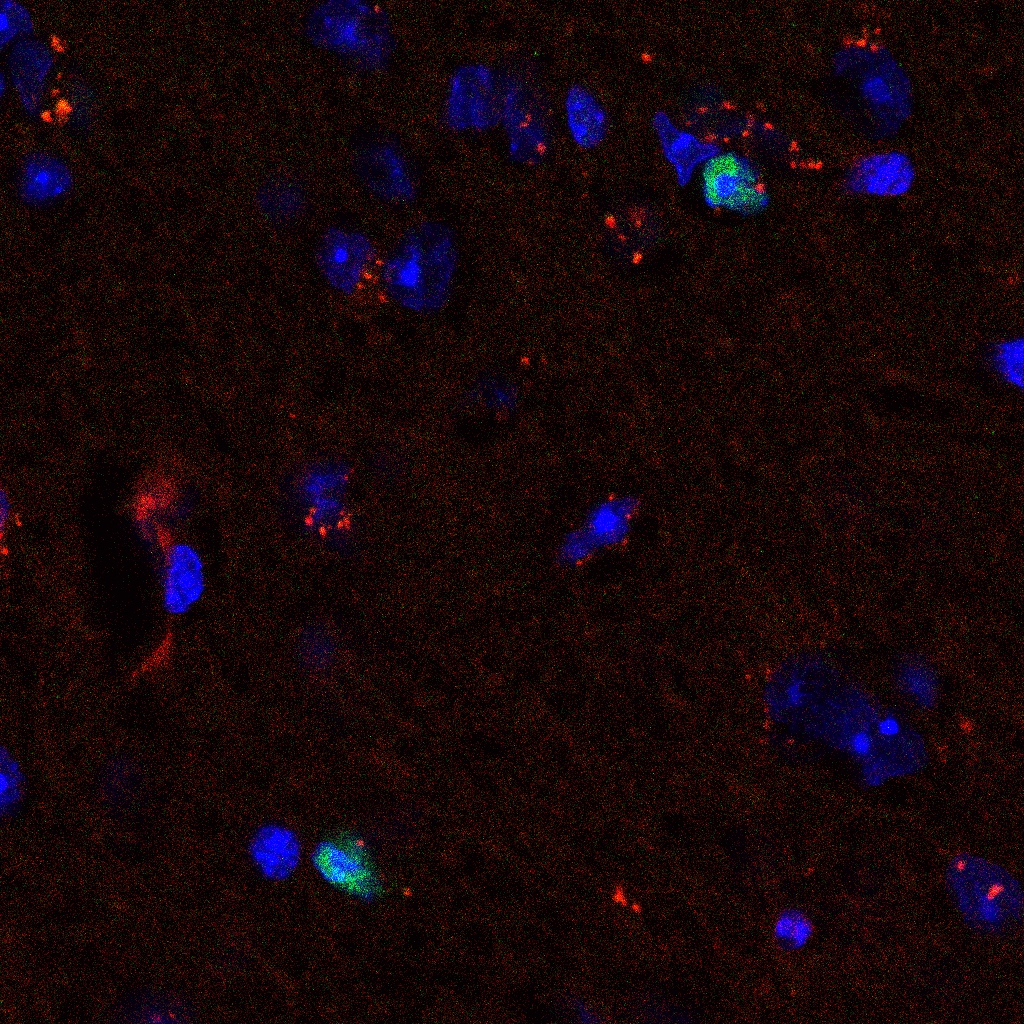

Supplement: Figure 8—source data 1. [file elife-82938-fig8-data1.zip › figure 8- source data 1/figure 8- source data 1_z11c1-3.jpg]

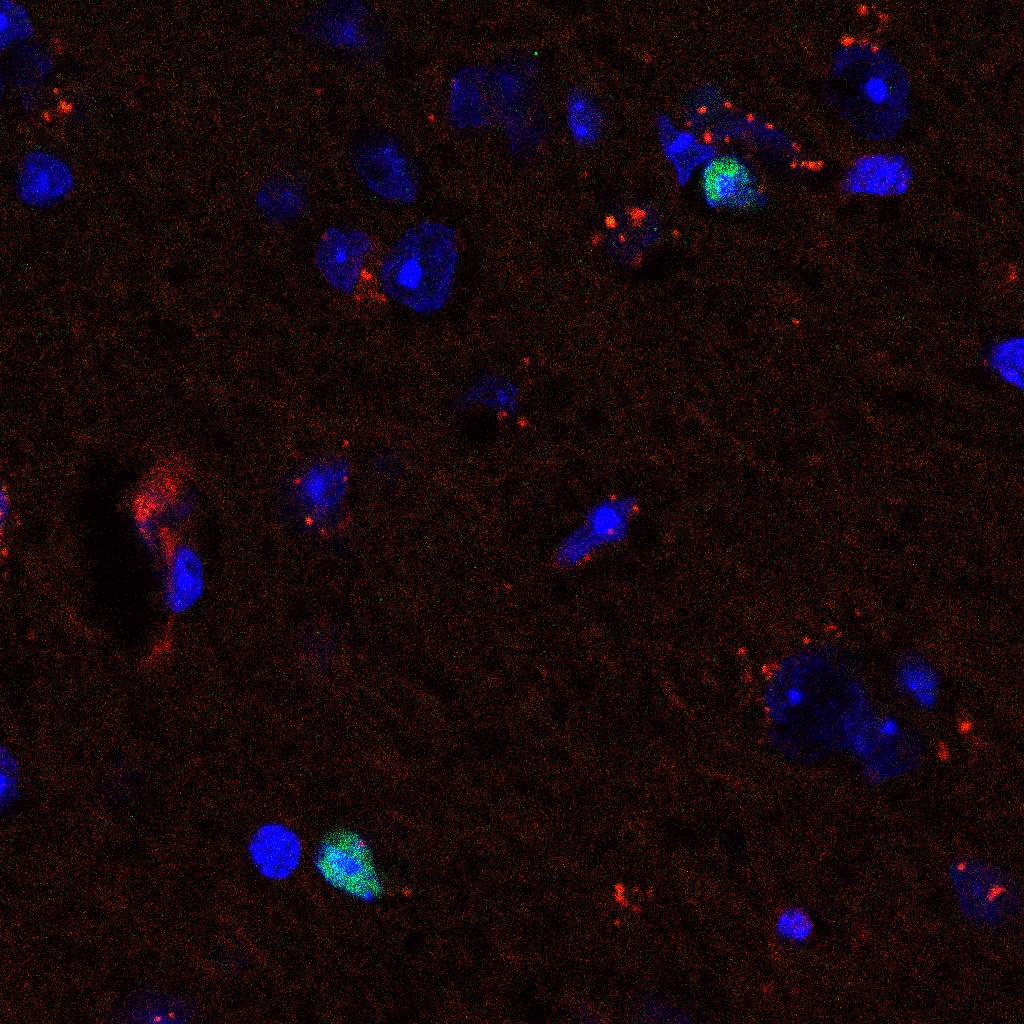

Supplement: Figure 8—source data 1. [file elife-82938-fig8-data1.zip › figure 8- source data 1/figure 8- source data 1_z12c1-3.jpg]

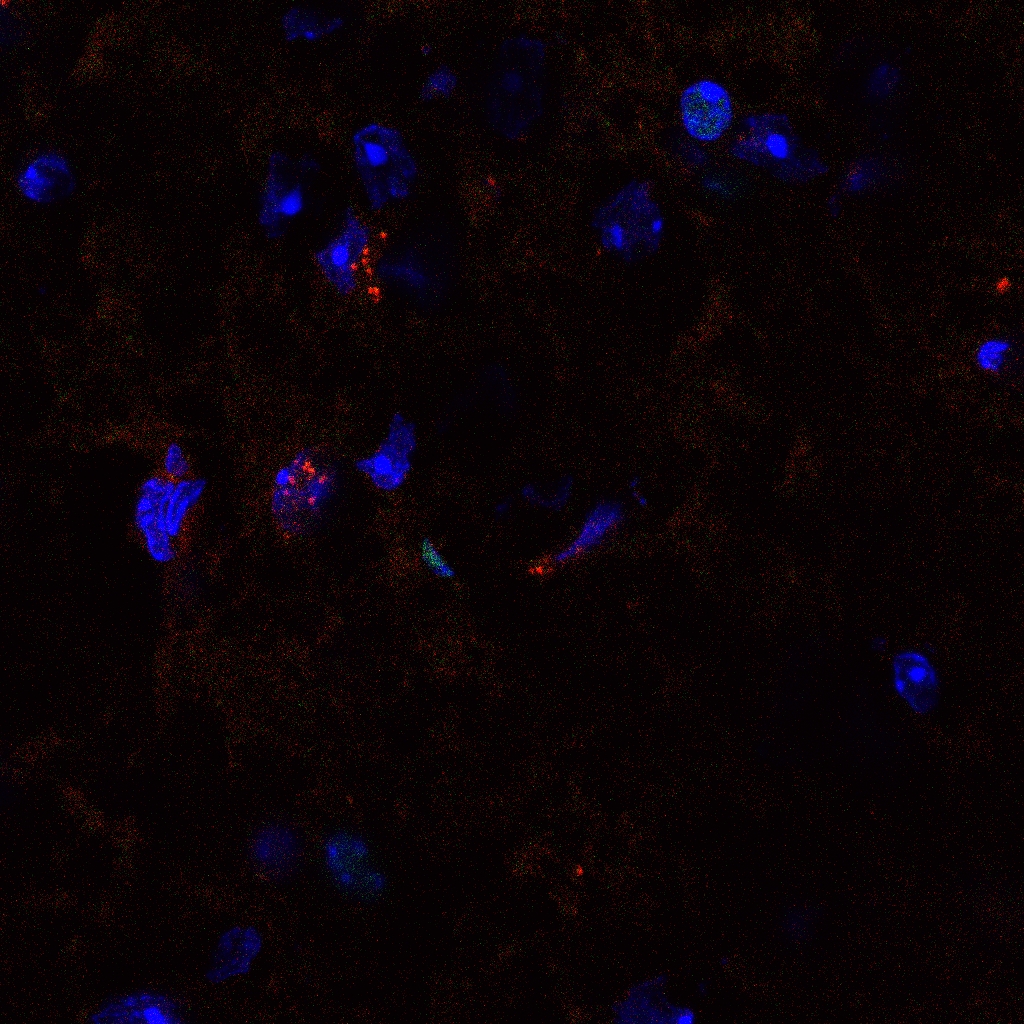

Supplement: Figure 8—source data 1. [file elife-82938-fig8-data1.zip › figure 8- source data 1/figure 8- source data 1_z18c1-3.jpg]
